# An ACE2-Fc decoy produced in glycoengineered plants neutralizes ancestral and newly emerging SARS-CoV-2 variants and demonstrates therapeutic efficacy in hamsters

**DOI:** 10.1038/s41598-025-95494-w

**Published:** 2025-04-02

**Authors:** Esther Föderl-Höbenreich, Shiva Izadi, Lara Hofacker, Nikolaus F. Kienzl, Alexandra Castilho, Richard Strasser, Ferran Tarrés-Freixas, Guillermo Cantero, Núria Roca, Mònica Pérez, Cristina Lorca-Oró, Carla Usai, Joaquim Segalés, Júlia Vergara-Alert, Lukas Mach, Kurt Zatloukal

**Affiliations:** 1 Diagnostic and Research Institute of Pathology, Medical University of Graz, Graz, Austria; 2 Department of Biotechnology and Food Sciences, Institute of Plant Biotechnology and Cell Biology, BOKU University, Vienna, Austria; 3 IRTA, Animal Health, Centre de Recerca en Sanitat Animal (CReSA), Campus de la Universitat Autònoma de Barcelona (UAB), 08193 Bellaterra, Catalonia Spain; 4 Unitat mixta d’investigació IRTA-UAB en Sanitat Animal, Centre de Recerca en Sanitat Animal (CReSA), Campus de la Universitat Autònoma de Barcelona (UAB), 08193 Bellaterra, Catalonia Spain; 5 Departament de Sanitat i Anatomia Animals, Facultat de Veterinària, Universitat Autònoma de Barcelona, 08193 Bellaterra, Catalonia Spain

**Keywords:** SARS-CoV-2, Antiviral, ACE2, Plant-based expression platform, Infectious diseases, Respiratory tract diseases, Plant biotechnology, Pathogens, Virology

## Abstract

Newly emerging SARS-CoV-2 variants of concern (VOCs) continue to drive COVID-19 waves and are typically associated with immune escape and increased resistance to current therapeutics including monoclonal antibodies. By contrast, VOCs still display strong binding to the host cell receptor ACE2. Consistent with these properties, we have now found that a soluble ACE2-Fc decoy produced in glycoengineered plants effectively neutralizes different SARS-CoV-2 isolates and exhibits even increased potency against VOCs as compared to an ancestral virus strain. In a golden Syrian hamster model, therapeutic intranasal delivery of ACE2-Fc effectively reduced weight loss and SARS-CoV-2 replication in the lungs when administered 24 h post-inoculation. This protective effect was not observed upon treatment of the infected animals with a non-binding ACE2-Fc mutant, demonstrating that the plant-derived ACE2-Fc decoy interferes specifically with the attachment of the virus to host cells. The results obtained provide support for further development of decoy-based antiviral approaches by plant molecular pharming.

**Supplementary Information:**

The online version contains supplementary material available at 10.1038/s41598-025-95494-w.

## Introduction

Since the outbreak of the COVID-19 pandemic, the evolution of SARS-CoV-2 resulted in the emergence of several variants of concern (VOCs), possessing heightened viral properties associated with increased infectivity, transmissibility, and changes in clinical disease presentation^1^. Notably, VOCs like Omicron have developed mechanisms to efficiently escape the immune system by partially evading neutralizing antibodies generated through vaccination and previous infections^2^. Although COVID-19 hospitalization rates have declined, SARS-CoV-2 remains a health threat to vulnerable groups such as infants, elderly individuals, or individuals with certain secondary medical conditions^3^.

SARS-CoV-2 is mainly transmitted through aerosols and initially infects the upper respiratory tract by binding to the ACE2 receptor on epithelial cells^4^. ACE2 is a highly glycosylated, membrane-anchored surface protein, expressed on a variety of cell types^5^. Mutations that enhance ACE2 affinity play a crucial role in the emergence of new SARS-CoV-2 variants by increasing viral transmission and infectivity^6^. However, the virus-neutralizing capacity of soluble ACE2 forms remains largely unaffected by viral escape mechanisms because increased affinity for host cell ACE2 simultaneously implies increased affinity for ACE2-based decoys^7^. Therefore, ACE2 mimics have attracted substantial interest as a promising therapeutic strategy.

Traditional production systems for recombinant therapeutic proteins, such as mammalian cell cultures, pose significant challenges, including high production costs, the necessity of sophisticated upstream facilities and the risk of potential product contamination with human pathogens. By contrast, plant-based expression platforms have emerged as versatile alternative production systems, in particular in the context of a rapid response to new epidemic threats^8^. Importantly, the availability of glycoengineered plant lines allows the production of recombinant proteins carrying human-like N-glycans^9^^,^ which are crucial for efficient binding of therapeutic antibodies to Fc receptors on immune cells^10^. Furthermore, human-like N-glycosylation is often required for proper protein folding and adequate pharmacokinetics^11^.

Recombinant proteins are frequently expressed as fusion proteins with the Fc domain of human IgG or other antibody classes, which provides several advantages. First, fusion to IgG Fc allows binding to the neonatal Fc receptor, providing an opportunity to escape lysosomal degradation and thus resulting in a prolonged half-life in the circulation^12,13^. Second, the Fc domain can be exploited for rapid and cost-effective purification via protein A affinity chromatography^14^. Third, the Fc domain can engage with Fc receptors on immune cells and thus promote cell-mediated and complement-dependent cytotoxicity, which can contribute to the elimination of virus-infected cells and the clearance of viral particles^15^. It has been shown that ACE2-Fc fusion proteins are highly effective in neutralizing SARS-CoV-2^16–19^.Furthermore, the circulatory half-life of ACE2 can be extended substantially by addition of IgG Fc^20^.

In a previous report, we have described the production of a fusion protein consisting of the protease domain of human ACE2 and the Fc domain of human IgG1 in glycoengineered *Nicotiana benthamiana* plants^21^*.* Here, we demonstrate the neutralization capacity of plant-derived ACE2-Fc against different VOCs of SARS-CoV-2 and further demonstrate its therapeutic activity in a golden Syrian hamster model.

## Results

### Plant-derived ACE2-Fc neutralizes a broad spectrum of SARS-CoV-2 variants of concern

**Fig. 1:**
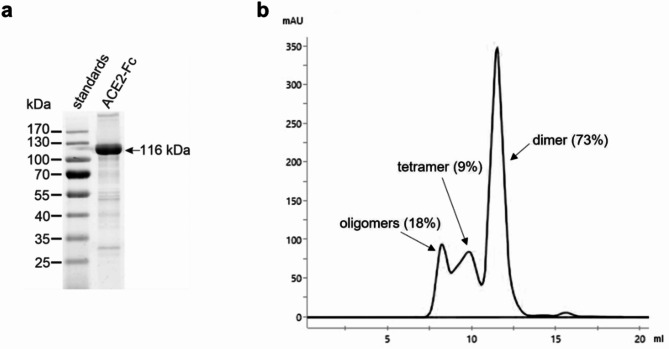
Characterization of ACE2-Fc produced in *Nicotiana benthamiana*. (**a**) Analysis of purified wild-type ACE2-Fc by SDS-PAGE under reducing conditions. (**b**) Fractionation of purified wild-type ACE2-Fc by size-exclusion chromatography on a Superdex 200 column. mAU, milli-absorbance units.

Wild-type human ACE2-Fc was isolated from agroinfiltrated *N. benthamiana* leaves by single-step affinity chromatography on immobilized protein A with yields of up to 650 µg per gram of leaf wet weight. Upon analysis by SDS-PAGE under reducing conditions, a major band of 116 kDa was observed (Fig. 1a; Supplementary Fig. 1). This agrees well with the theoretical molecular mass of the recombinant fusion protein (108 kDa without accounting for N-glycosylation). Consistent with Fc-mediated homodimerization, purified ACE2-Fc was largely in a dimeric state (73%). However, tetramers (9%) and higher oligomers (18%) were also detected by size-exclusion chromatography (Fig. 1b).

**Fig. 2:**
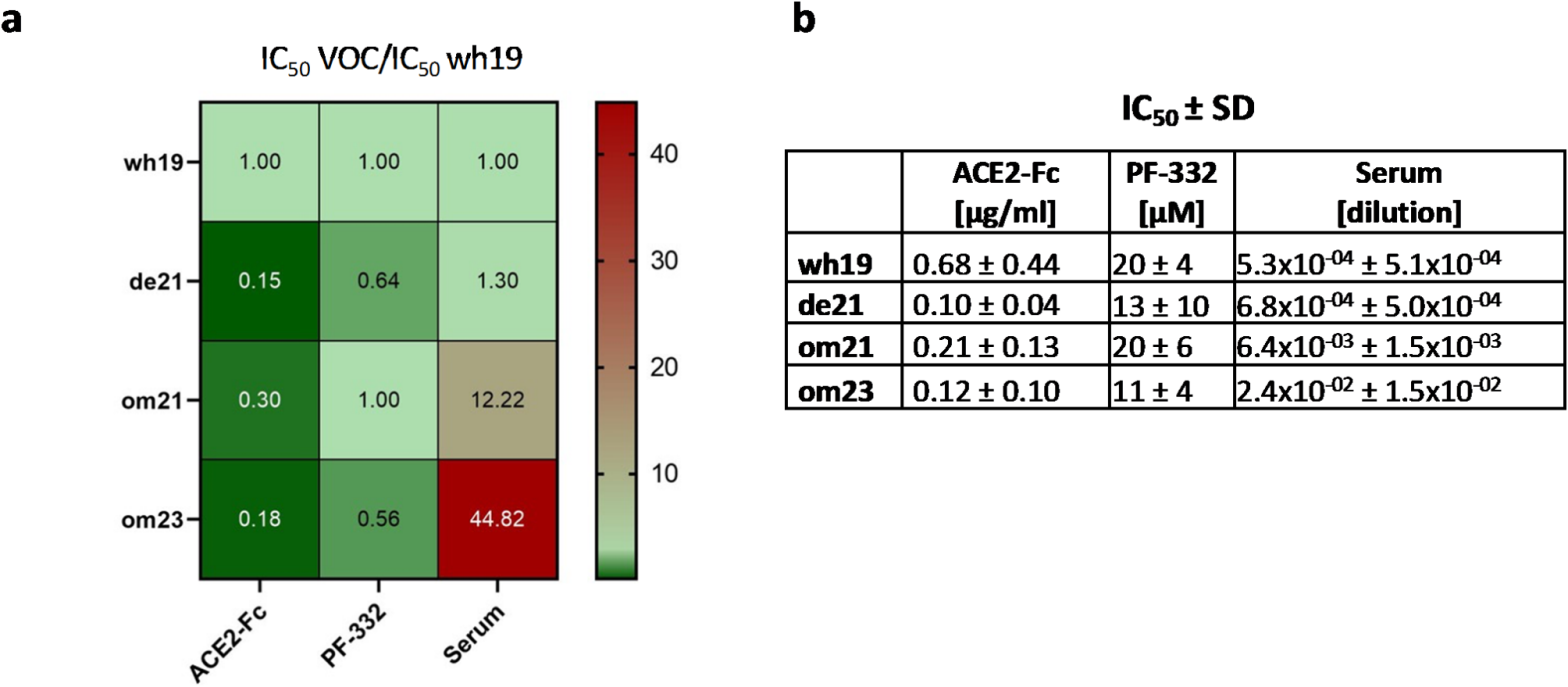
(**a**) Heat map depicting fold change of antiviral activity of wild-type ACE2-Fc, the viral main protease inhibitor (PF-332), and a pool of COVID-19 convalescent sera (serum) against a Wuhan variant (wh19), a Delta variant (de21) and two Omicron variants (om21, om23). IC_50_ values were determined by cytopathic effect inhibition assays in VeroE6 cells and inhibitory dose–response curve analysis. (**b**) IC_50_ values ± standard deviation (SD). Data refer to arithmetic mean values from two to three independent experimental series performed in triplicates.

To assess the neutralization potential of wild-type ACE2-Fc against SARS-CoV-2, we determined the half-maximal inhibitory concentration (IC_50_) of several antiviral agents and compared their activity against an ancestral SARS-CoV-2 variant (Wuhan; wh19) and three VOCs: a Delta variant (de21), an early Omicron isolate (om21; lineage B.1.1.529) and a more recent Omicron strain (om23; lineage EG5.1). Wild-type ACE2-Fc exhibited strong neutralization of all variants, with even increased neutralization activity against the later variants compared to the ancestral wh19 variant (Fig. 2). By contrast, COVID-19 convalescent sera showed reduced neutralization activity against the later variants. Nirmatrelvir, a viral main protease inhibitor (PF-332), displayed similar potency against all SARS-CoV-2 isolates tested, in line with its reported broad activity against VOCs^22^.

**Fig. 3:**
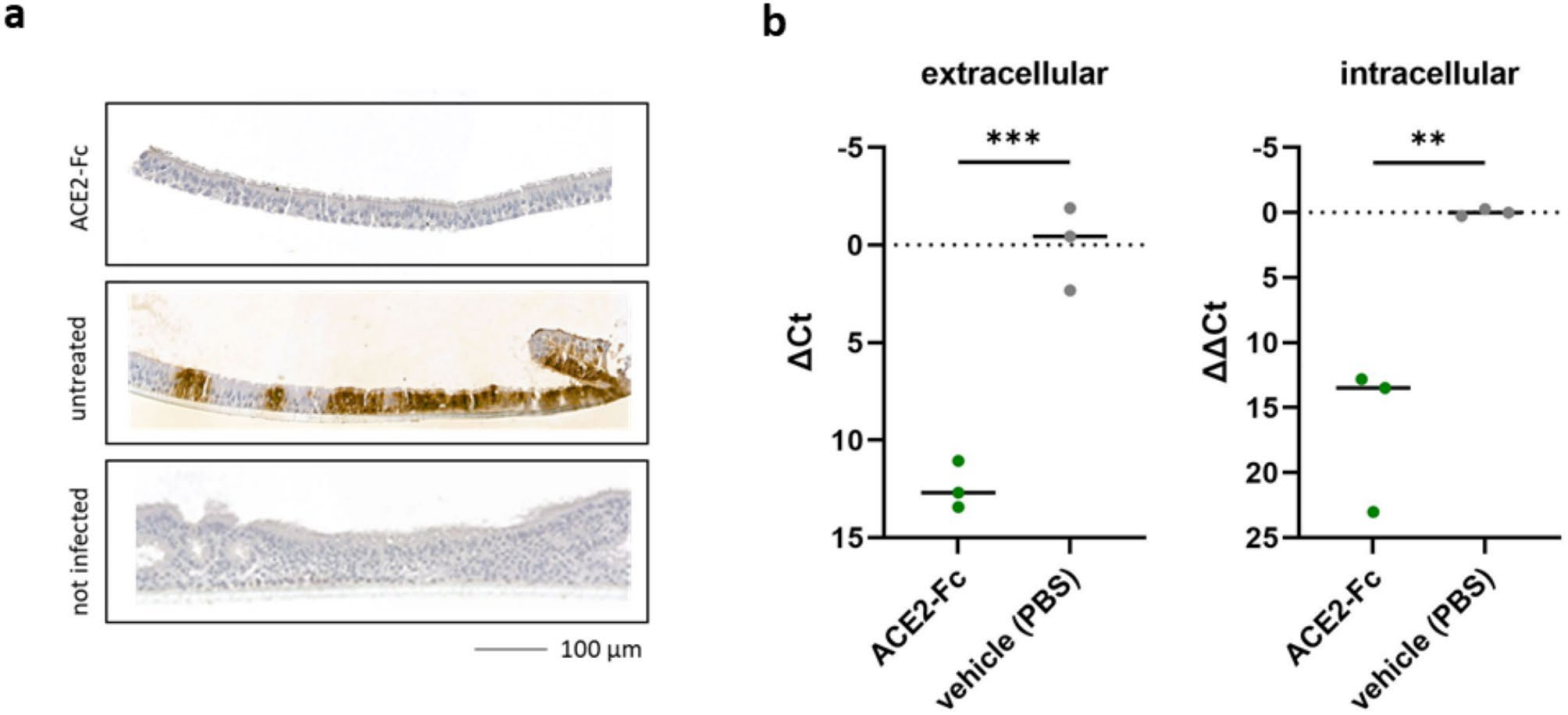
Wild-type ACE2-Fc prevents SARS-CoV-2 (de21) infection of primary air liquid interface cultures as shown by (**a**) immunohistochemical detection of the viral nucleocapsid protein and (**b**) detection of viral RNA by RT-qPCR. Extracellular SARS-CoV-2 N gene expression is presented as ∆Ct (Ct N_(infected, ACE2-Fc) _– Ct N_(infected, untreated)_). Intracellular SARS-CoV-2 N gene is expressed as ∆∆Ct ({Ct N_(infected, ACE2-Fc)_ – Ct HPRT1_(infected, ACE2-Fc)_} – {Ct N_(infected, untreated)_ – Ct HPRT1_(infected, untreated)_}). Dots represent independent cultures and bars the median values. *P* values were calculated by ANOVA and corrected for multiple comparison by Bonferroni. *P* values < 0.05 were considered significant (*P < 0.05; **P < 0.01; ***P < 0.001; ****P < 0.0001).

To evaluate the antiviral activity of wild-type ACE2-Fc in a 3D culture model that resembles the human respiratory epithelium, we used primary air liquid interface (ALI) cultures, which are considered physiologically relevant models for studying respiratory viral infections. We showed that pre-treatment of SARS-CoV-2 (de21) with 20 µg/ml wild-type ACE2-Fc efficiently inhibited infection of primary ALI cultures, as evidenced by immunohistochemical staining for the SARS-CoV-2 nucleocapsid protein as well as quantification of both intracellular and extracellular viral RNA (Fig. 3).

### Development of a non-binding ACE2-Fc mutant as a control for in vivo studies

**Fig. 4:**
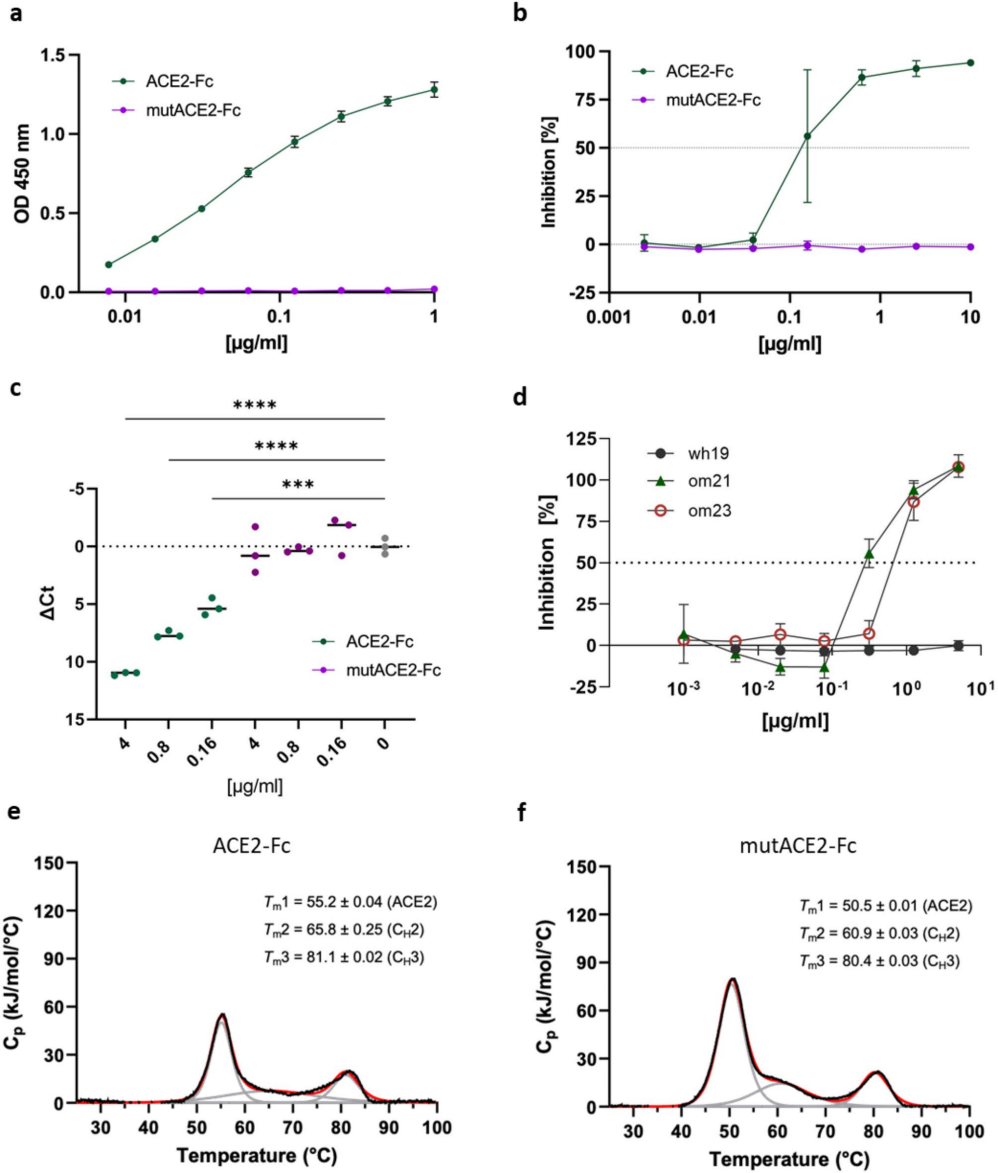
(**a**) Binding affinity of wild-type ACE2-Fc and mutACE2-Fc for immobilized Wuhan RBD assessed by ELISA. Data shown represent arithmetic mean values ± standard deviation (SD) of one representative experiment performed in technical duplicates. This experiment was repeated two times with similar results. (**b**) Dose response curve of wild-type ACE2-Fc and mutACE2-Fc against SARS-CoV-2 (de21) in VeroE6 cells assessed by cytopathic effect assays. Data are presented as arithmetic mean values ± SD of one experiment performed in triplicates. (**c**) Inhibition of SARS-CoV-2 (de21) replication was assessed by quantification of viral RNA in VeroE6 supernatants at 24 h post-infection by RT-qPCR. Extracellular SARS-CoV-2 N gene expression is presented as ∆Ct (Ct N_(infected, treated)_ – Ct N_(infected, untreated_)). Dots represent median values of one experiment performed in triplicates. *P*-values were calculated by ANOVA followed by Bonferroni post hoc test. *P* values < 0.05 were considered significant (* *P* < 0.05; ** *P* < 0.01; *** *P* < 0.001; ****P < 0.0001). (**d**) Dose response curve of mutACE2-Fc against different SARS-CoV-2 variants (wh19, om21, om23) in VeroE6 cells assessed by cytopathic effect assays. Data are presented as arithmetic mean values ± SD of one experiment performed in triplicates. (**e**,**f**) Analysis of wild-type ACE2-Fc (**e**) and mutACE2-Fc (**f**) by differential scanning calorimetry. Raw data (black) were smoothened (red) and then fitted using a non-two-state thermal unfolding model (gray). Data are presented as mean ± SEM of three independent experiments. Cp, heat capacitance.

To demonstrate the specificity of the antiviral activity of wild-type ACE2-Fc, we designed a mutated ACE2-Fc variant (mutACE2-Fc). In mutACE2-Fc, eight residues of human ACE2 (Q24; D30; K31; H34; L79; M82; Y83; K353) forming contacts with the receptor-binding domain (RBD) of the SARS-CoV-2 spike protein were replaced with their mouse ACE2 counterparts (N24; N30; N31; Q34; T79; S82; F83; H353)^18^. It has been shown that mouse ACE2 does not bind to the RBD of Wuhan and Delta strains, although its affinity to the RBD of Omicron BA.1 is comparable to that of human ACE2^23^. Indeed, mutACE2-Fc lost the capacity of the wild-type protein to bind to Wuhan RBD in enzyme-linked immunosorbent assays (Fig. 4a) and failed to protect VeroE6 cells against infection by the Delta strain used in our studies (Fig. 4b,c). By contrast, we detected residual neutralizing activity against our Omicron isolates (Fig. 4d). It is of note that the introduction of the eight mutations reduced the stability of the ACE2 and C_H_2 domains of mutACE2-Fc to some extent, as determined by differential scanning calorimetry (Fig. 4e,f). However, the enzymatic activity of mutACE2-Fc (18.6 ± 0.3 units per mg protein) was comparable to that of wild-type ACE2-Fc (17.1 ± 0.4 units per mg protein). Taken together, these findings qualify mutACE2-Fc as a negative control for in vivo studies.

### Intranasal application of plant-derived ACE2-Fc reduces weight loss of SARS-CoV-2-infected hamsters

**Fig. 5:**
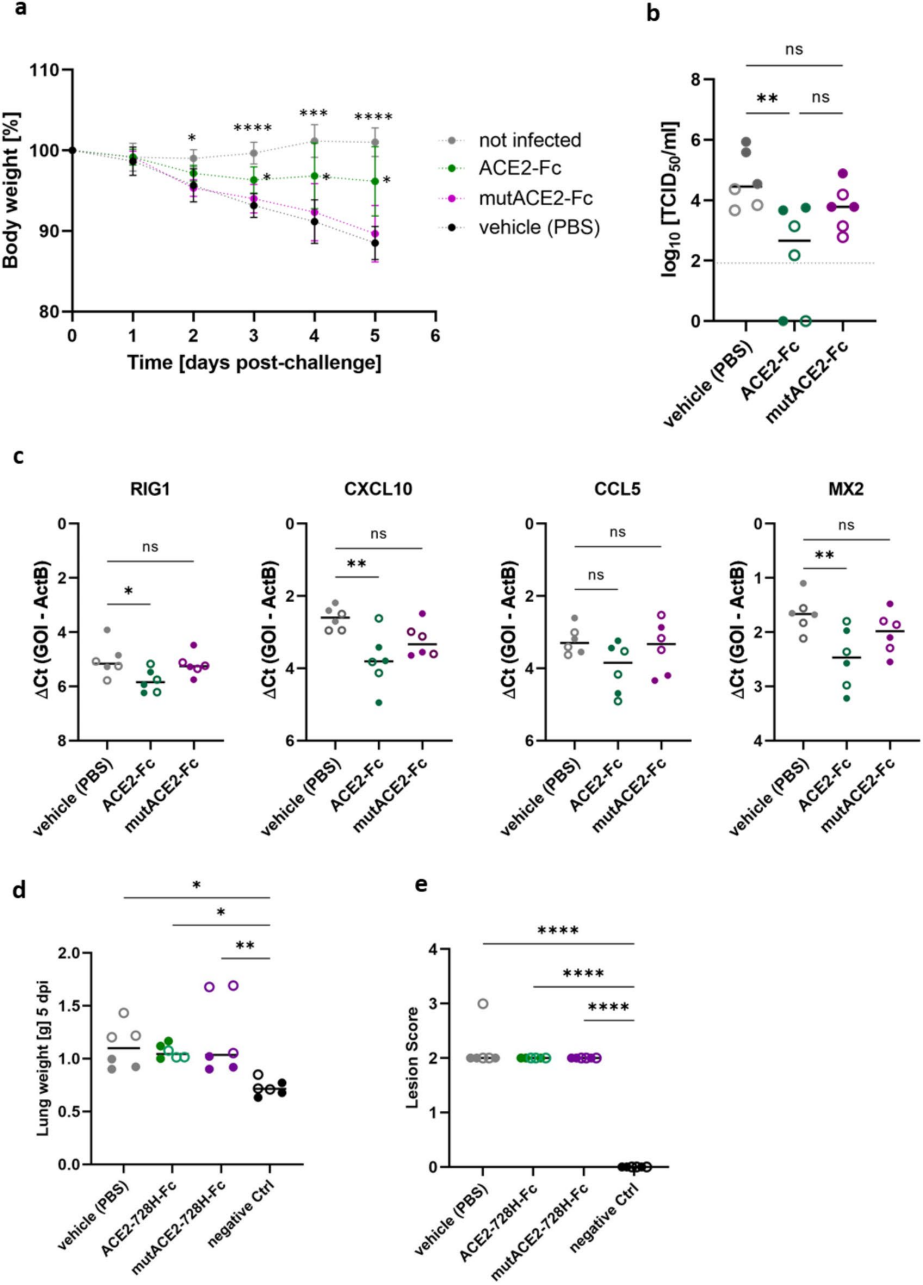
(**a**) Body weight variation relative to the day of infection. Data are shown as mean ± SD (n = 6). *P*-values were determined by ANOVA with matched values followed by Bonferroni post hoc test. Asterisks indicate significant differences to vehicle-treated animals. (**b**) Infectious virus loads in lungs were determined at 5 dpi and analysed by ANOVA followed by Bonferroni post hoc test. Full circles represent males, empty circles female animals (6 animals per group; n = 3 for each sex). (**c**) Expression levels of inflammatory cytokines and chemokines in lungs as quantified by RT-qPCR. (**d**) Lung weights at 5 dpi. (**e**) Histopathological assessment of lungs 5 dpi. Lung damage was semi-quantitatively scored based on the level of inflammation (0: no; 1: mild; 2: moderate; 3: severe). Data were analysed using ANOVA followed by Bonferroni post hoc test. Bars represent median values. *P* values < 0.05 were considered significant (*P < 0.05; **P < 0.01; ***P < 0.001; ****P < 0.0001). ns, not significant (*P* > 0.05). GOI, gene of interest.

Based on our in vitro findings, we performed an in vivo study in golden Syrian hamsters to evaluate the therapeutic potential of plant-derived ACE2-Fc. To simulate a therapeutic setting, hamsters were infected with a Wuhan D614G (S) strain of SARS-CoV-2 (hCoV-19/France/GE1973/2020, 10^4^ TCID_50_/ml, 50 µl/nostril), and received daily intranasal administrations (50 µl/nostril) of either 2.5 mg/ml wild-type ACE2-Fc, 2.5 mg/ml mutACE2-Fc, or vehicle (phosphate-buffered saline, PBS), starting 24 h after infection. On the day of euthanasia (day 5 post-infection, 5 dpi), hamsters treated with wild-type ACE2-Fc showed a significantly lower average body weight loss (3.9%) than vehicle-treated animals (11.5%) as compared to uninfected controls. The group receiving mutACE2-Fc showed a similar weight loss (10.3%) as the vehicle group (Fig. 5a). Additionally, treatment with wild-type ACE2-Fc resulted in a significant reduction of the amount of infectious virus particles present in the lungs of the hamsters (2.5 log_10_ decrease, Fig. 5b) and concomitantly in decreased expression levels of pro-inflammatory cytokines and chemokines (Fig. 5c). However, lung weights were significantly higher in all groups compared to uninfected hamsters (Fig. 5d). Additionally, we observed no improvement in histopathological lung inflammation scores in the ACE2-Fc treated animals (Fig. 5e).

**Fig. 6:**
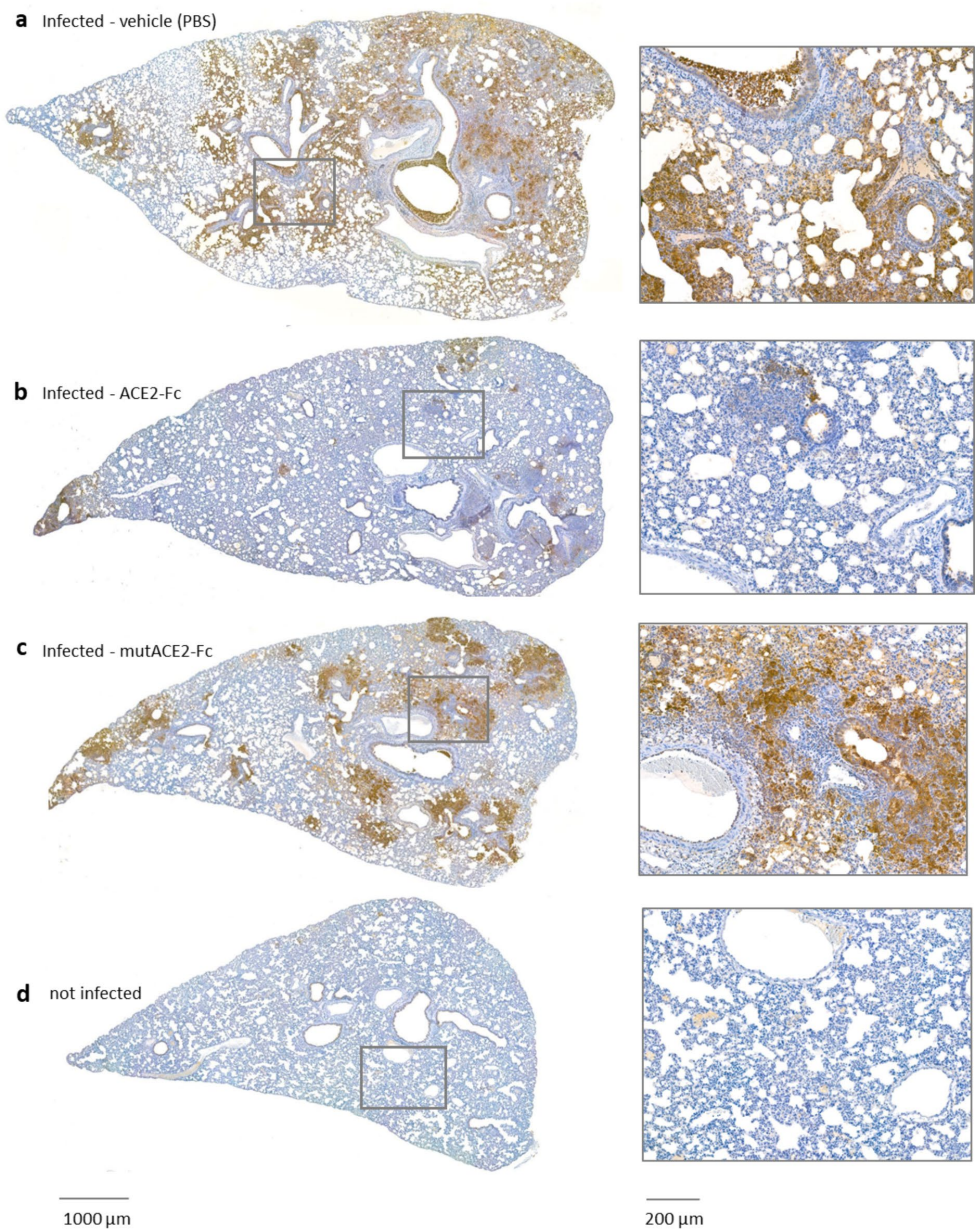
Immunohistochemical detection of SARS-CoV-2 nucleocapsid protein in lung tissues 5 days after infection. Lungs from animals treated daily with vehicle (**a**), wild-type ACE2-Fc (**b**) or mutACE2-Fc (**c**). Lung tissue of an uninfected animal (**d**) was used as a negative control. Representative sections are shown in two magnifications.

Strikingly, immunohistochemical staining of lung sections with antibodies to the SARS-CoV-2 nucleocapsid protein revealed that repeated post-infection treatment with wild-type ACE2-Fc led to a marked reduction in the number of virus-infected cells, whereas the extent of SARS-CoV-2 infection in the lungs of hamsters treated with mutACE2-Fc was similar as in the vehicle-treated group (Fig. 6). The corresponding hematoxylin/eosin stained sections are shown in Supplementary Fig. 2.

## Discussion

The continuous evolution of SARS-CoV-2 drives the emergence of new variants including VOCs, which are naturally selected for improved viral spread. These variants often exhibit mutations that increase the affinity for the entry receptor ACE2, allow escape from immune recognition, or confer resistance to antiviral drugs. As a result, many monoclonal antibodies initially used for COVID-19 therapy^24^ and convalescent antibodies induced by vaccination or previous infections have become less effective in recognizing viral epitopes^25^. Thus, the development of alternative therapeutic approaches, which are effective against newly emerging VOCs, are of great relevance. Generally, VOCs are characterized by their maintained or even enhanced affinity for ACE2^6,26,27^. For this reason, the neutralizing capacity of ACE2-based decoys remains largely unaffected by viral escape mechanisms because increased affinity for host cell ACE2 simultaneously implies increased affinity for ACE2-based decoys. In this study, we have demonstrated that an ACE2-Fc fusion protein produced in glycoengineered plants can effectively neutralize several VOCs and has therapeutic activity in a golden Syrian hamster model of SARS-CoV-2 infection.

Upon infection with SARS-CoV-2, golden Syrian hamsters develop a lung pathology resembling human COVID-19 pneumonia, accompanied by moderate weight loss serving as a primary indicator of disease severity^28,29^. Notably, later evolved variants like Omicron generally do not lead to severe disease phenotypes or weight loss in hamsters^30^. Therefore, we used an ancestral variant to allow phenotypic monitoring. Importantly, the capacity of ACE2-Fc to neutralize a broad spectrum of VOCs, including Omicron variants, was confirmed in vitro (Fig. 2), suggesting that our in vivo findings are also relevant to current and emerging VOCs.

The concept of soluble ACE2 decoys has been investigated in hamsters by several research groups following different strategies regarding decoy designs and study protocols^16,31–37^. Most previous studies administered ACE2 decoys within two to twelve hours post-infection, targeting early stages of infection^16,32–34^. By contrast, the first dose of ACE2-Fc was administered in our study at 24 h post-infection to examine its therapeutic effect on the hamsters at a later time point when viral replication and disease progression were already ongoing. Indeed, we found that daily intranasal administration of 250 µg ACE2-Fc significantly reduced SARS-CoV-2 replication, weight loss and lung infection (Fig. 5), but this did not prevent lung damage and edema. Liu and colleagues^31^ reported a 3-log10 reduction in lung virus titers and 4% less body weight loss when hamsters were treated with a daily lung-delivered dose of 4 mg HH-120 (a mixture of pentameric and hexameric ACE2-Fc) per kg body weight. These findings align well with our data of a 2-log10 reduction of the virus load in lungs and 8% less body weight loss upon daily intranasal application of 2.5 mg ACE2-Fc per kg body weight.

ACE2-Fc comprises a human IgG1 Fc-domain, which can be recognized by Fc receptors (FcRs) on immune cells and therefore could potentially contribute to viral clearance. Interestingly, Torchia et al.^34^ reported higher beneficial effects of ACE2-Fc decoys capable of inducing FcR functions when compared to decoys with abrogated FcR binding capacity. Nevertheless, there is an ongoing debate regarding potential FcR-mediated side effects of Fc fusion proteins^14,38^. As a control for unspecific Fc-induced effects, we used a mutated ACE2-Fc decoy (mutACE2-Fc) unable to neutralize the ancestral Wuhan strain of SARS-CoV-2 used in our hamster experiment. This non-binding ACE2-Fc variant was created by substituting 8 amino acids engaged in the interaction between human ACE2 and the RBD of the SARS-CoV-2 spike protein with their mouse ACE2 counterparts, because wild-type mice were found to be resistant to infection by early SARS-CoV-2 variants^39^. We used mutACE2-Fc to demonstrate the specific mode of action of wild-type ACE2-Fc in hamsters infected with the Wuhan strain of SARS-CoV-2. However, the use of mutACE2-Fc as a specificity control may be limited to studies involving the Wuhan variant and early VOCs such as Delta since recent studies found that Omicron variants have acquired the ability to infect mice^40,41^. This is in good agreement with the observation that mutACE2-Fc can neutralize our Omicron strains om21 and om23 (Fig. 4d). Therefore, mutACE2 might not be a suitable control for hamster experiments with newly emerging SARS-CoV-2 variants.

Several reports have demonstrated that human ACE2-Fc and other ACE2 decoys can be produced in plant-based expression platforms in good yield and purity^21,36,42–45^. While these decoys were all capable of neutralizing SARS-CoV-2 in cell-based assays, only one construct was also tested in a hamster experiment. In this study^36^, the animals received intraperitoneal injections of 2.5 mg per kg body weight of ACE2-Fd (a fusion protein consisting of the human ACE2 ectodomain and the T4 fibritin trimerization foldon) on days 1–5 after intranasal infection with SARS-CoV-2. While treatment with ACE2-Fd resulted in a slight reduction of the weight loss experienced by the infected hamsters, the amount of infectious virus particles in the lungs of the animals remained unchanged. The much stronger protective effect of ACE2-Fc in our hamster experiment suggests that intranasal application of ACE2 decoys leads to a particularly pronounced interference with SARS-CoV-2 replication in the respiratory tract.

In conclusion, our data highlight the potential of plant-produced ACE2-Fc decoys with human-like glycosylation to be used for the development of antiviral drug candidates with a broad neutralization spectrum against present and probably also future SARS-CoV-2 VOCs. Hence, we expect that plant-derived ACE2-Fc decoys can be also considered as treatment options for other betacoronaviruses which use ACE2 as host cell receptor.

## Material and methods

### Ethics declaration and biosafety statements

The animal experiment was approved by the Institutional Animal Welfare Committee of the Institut de Recerca i Tecnologia Agroalimentàries (CEEA-IRTA, registration number CEEA 365/2023) and by the Ethical Commission of Animal Experimentation of the Autonomous Government of Catalonia (registration number CEA-OH/12069/1) and conducted by certified staff. Experiments with SARS-CoV-2 were performed at the Biosafety Level-3 (BSL-3) facilities of the Biocontainment Unit of IRTA-CReSA (Barcelona, Spain) under the approval of the biosafety committee (registration number CBS 116/2023). Animal studies were carried out in compliance with the ARRIVE guidelines and in accordance with relevant guidelines and regulations.

Convalescent sera were collected from adult volunteers after written informed consent at the Medical University of Graz and were approved by the ethics committee of the Medical University of Graz (EK number: 34-203 ex 21/22). All research involving human samples was performed in accordance with the Declaration of Helsinki and the Research Organisation Act, the Federal Act on Hospitals and Sanatoria and the Clinical Trials Regulation.

### Production of ACE2-Fc in glycoengineered plants

Wild-type and mutant ACE2-Fc proteins encompassing ACE2 residues 18-728 were produced as described previously for other ACE2-Fc variants^21,45^. Briefly, recombinant ACE2-Fc fusion proteins were transiently expressed in leaves of glycoengineered *N. benthamiana* ΔXT/FT plants^46^ by agrobacterium-mediated transfection. Batches of 30 g leaf wet weight were extracted and then purified by affinity chromatography using a protein A column, resulting in the isolation of up to 20 mg ACE2-Fc per batch. In contrast to previous studies^21,45^, affinity-purified ACE2-Fc was not subjected to a final polishing step by size exclusion chromatography.

### Enzyme-linked immunosorbent assays

Enzyme-linked immunosorbent assays were carried out according to standard protocols. Briefly, 96-well plates (Nunc MaxiSorp; Thermo Fisher Scientific, Waltham, MA, USA) were coated overnight at 4 °C with 200 ng per well of in-house produced Wuhan RBD^47^ in 100 µl PBS. Plates were then washed three times with PBS containing 0.05% Tween 20 (PBST). From an initial concentration of 1 µg/ml, twofold serial dilutions of the ACE2-Fc samples were prepared in PBST containing 1% bovine serum albumin (dilution buffer), and 50 µl then added per well. After incubation for 1 h at 37 °C, the plates were washed three times with PBST prior to addition of 50 µl of 0.03 µg/ml peroxidase-conjugated goat antibodies to human Fc (Sigma-Aldrich, St. Louis, MO, USA) in dilution buffer prior to incubation for 1 h at 37 °C. After washing of the plates as above, bound peroxidase activity was detected by addition of 100 µl of ELISA substrate solution (0.1 mg/ml tetramethylbenzidine (Sigma-Aldrich) in 100 mM citric acid/sodium phosphate buffer (pH 5.0) containing 0.006% H_2_O_2_). After 5–15 min, the reaction was stopped by addition of 100 µl 0.18 M H_2_SO_4_ prior to measurement of the optical density at 450 nm (reference wavelength: 620 nm) using a Spark multi-channel spectrophotometer (Tecan, Grödig, Austria).

### Differential scanning calorimetry

Differential scanning calorimetry experiments were performed as described^17^ using a MicroCal PEAQ-DSC Automated system (Malvern Panalytical, Malvern, UK), using 5–10 µM protein solutions in PBS. The heating was performed from 20 to 100 °C at a rate of 1 °C/min. The protein solution was then cooled in situ and an identical thermal scan was run to obtain the baseline for subtraction from the first scan. All measurements were performed in triplicates. Fitting was done with Origin 7.0 for DSC software using the non-2-state transition model.

### ACE2 activity assays

The enzymatic activity of wild-type and mutant ACE2-Fc was determined using 50 µM 7-methoxycoumarin-4-yl-acetyl-Ala-Pro-Lys-2,4-dinitrophenyl (Bachem, Bubendorf, Switzerland) as substrate. Assays were performed in 50 mM MES buffer (pH 6.5) containing 300 mM NaCl, 10 µM ZnCl_2_ and 0.01% Brij-35^48^ in a final volume of 200 µl. After addition of 10 ng ACE2-Fc (appropriately diluted in assay buffer containing 0.1% bovine serum albumin) and incubation for 5 min at ambient temperature, reactions were stopped by addition of 1 ml 50 mM EDTA prior to analysis by spectrofluorimetry (excitation wavelength: 320 nm,emission wavelength: 405 nm). A standard curve was generated with 1–5 µM completely hydrolysed substrate. One unit of enzymatic activity corresponds to 1 µmol of substrate hydrolysed per min.

### Cells

African green monkey kidney epithelial cells (VeroE6) were obtained from Biomedica (Vienna, Austria) and grown in Minimal Essential Medium (MEM) supplemented with Earle’s Salts and L-Glutamine (Thermo Fisher Scientific), 1% Penicillin–Streptomycin stock solution (PenStrep) and 2% fetal bovine serum (Thermo Fisher Scientific). Primary human air liquid interface cultures (MucilAir™) were purchased from Epithelix (Plan-les-Ouates, Switzerland) and cultured according to the manufacturer’s instructions.

### Viruses

All experimental procedures with SARS-CoV-2 were performed in a BSL-3 laboratory. SARS-CoV-2 Wuhan-Hu-1 (wh19: Human 2019-nCoV Isolate, Ref-SKU 026V-03883), as disclosed by the Center for Disease Control and Prevention, was purchased from EVAg. The Omicron isolate 2021 (om21), lineage B.1.1.529 was also purchased from EVAg (SKU: 010V-04425). The Delta isolate (de21) and the Omicron isolate 2023 (om23; lineage EG5.1) were isolated at the Medical University of Graz in July 2021 and September 2023, respectively. Virus variants were propagated in VeroE6 cells at 37 °C and 5% CO_2_ for 72–96 h. Prior to harvesting, cells were lysed by a freeze–thaw cycle, followed by a centrifugation step (10 min, 3000 × g) to remove cellular debris. Supernatants were filtered with 0.2 µm syringe filters (Thermo Fisher Scientific) and virus stocks were stored at − 80 °C until use. To determine viral titers, confluent VeroE6 cultures were infected with serial tenfold dilutions of virus stocks. After 96 h of incubation at 37 °C and 5% CO_2_ cells were fixed with 4% neutral-buffered formalin and cytopathic effects were visualized by crystal violet staining. TCID_50_ values were calculated by the Reed-Muench method^49^. All virus stocks used in this study were sequenced as described elsewhere^50^. Sequences are provided in the supplementary section (Supplementary Table 1). The virus strain used for the in vivo experiment was hCoV-19/France/GE1973/2020, clade G, D614G (S) supplied by the National Reference Centre for Respiratory Viruses hosted by Institut Pasteur (Paris, France).

### Virus neutralization assays

The viral main protease inhibitor PF-07321332 (PF-332) was purchased from Selleckchem (Catalog No. S9866). Convalescent sera from 4 donors were collected at the Medical University of Graz between August 2021 and July 2022. Sera were heat-inactivated and pooled for neutralization assays. Neutralizing activity was assessed by cytopathic effect assays, as described previously^51^. Briefly, VeroE6 cells were seeded in 96-well plates at a density of 1 × 10^4^ per well. Antiviral compounds were serially diluted (fourfold) and incubated with SARS-CoV-2 for 15 min at 37 °C. Subsequently, decoy-virus mixtures were added to VeroE6 cells in triplicates and incubated for 96 h at 37 °C. Cells were fixed with 4% neutral-buffered formalin and cytopathic effects were assessed by crystal violet staining. The stained cells were dissolved in 10% acetic acid prior to measuring the optical density of the samples at 595 nm in a plate reader. Data were normalized to a no-decoy control (0% inhibition) and a no-virus control (100% inhibition). IC_50_ values were calculated by nonlinear regression analysis with variable slopes in GraphPad Prism version 9.

Primary human ALI cultures (MucilAir, Epithelix) were washed once with PBS prior to infection and the basal medium was replenished with MucilAir culture medium containing 20 µg/ml ACE2-Fc. 5 × 10^3^ pfu SARS-CoV-2 (de21) were incubated with ACE2-Fc (20 µg/ml) and subsequently added to the apical side of the ALI culture. After 1 h incubation at 37 °C, the virus inoculum was removed, and the apical side was washed three times with PBS to remove residual extracellular virus. Virus release was assessed 24 h post-infection (hpi) by washing the apical side with 200 µl PBS (apical wash). Viral RNA was then isolated from the apical wash using the QiaAmp Viral RNA Minikit (Qiagen, Germany), according to the manufacturer’s protocol. SARS-CoV-2 replication was quantified via RT-qPCR using the QuantiTect Multiplex RT-qPCR Kit (Qiagen) with a Rotor Gene Q cycler (Qiagen). The reactions were performed in a total volume of 25 µL at 50 °C for 30 min followed by 95 °C for 15 min and 45 cycles of 95 °C for 3 s and 55 °C for 30 s. Forward primer: 2019-nCoV_N1-F 5′-GACCCCAAAATCAGCGAAAT-3′; reverse primer: 2019-nCoV_N1-R 5′-TCTGGTTACTGCCAGTTGAATCTG-3′; probe: 2019-nCoV_N1-P 5′-FAM-ACCCCGCATTACGTTTGGTGGACC-BHQ1-3′.

Transwell membranes were cut in half for intracellular virus quantification and immunohistochemical analysis. Intracellular virus RNA was isolated using the RNeasy Mini Kit (Qiagen). Viral RNA was quantified by two-step RT-qPCR using LunaScript RT SuperMix (New England Biolabs, Frankfurt, Germany) and Luna Universal qPCR Master Mix (New England Biolabs) together with the N1 primer pair mentioned above. HPRT1 was used as housekeeping gene (primers: 5′-TCAGGCAGTATAATCCAAAGATGGT-3′; 5′-AGTCTGGCTTATATCCAACACTTCG-3′).

### In vivo experiment

A total of thirty-six 6- to 8-week-old male and female golden Syrian hamsters (Charles River Laboratories, Wilmington, MA, USA) were divided into 6 groups (6 animals/group). Animals were inoculated with SARS-CoV-2 as described previously^52^ and then treated on 4 consecutive days with either 2.5 mg/ml wild-type ACE2-Fc, 2.5 mg/ml mutACE2-Fc or vehicle (PBS) alone. Non-infected animals were treated on 4 consecutive days with 2.5 mg/ml ACE2-Fc plus 60 µM hexahydroxystilbene^53^ in PBS. Briefly, animals were intranasally inoculated with 10^4^ TCID_50_ per animal (100 µL total, 50 µL/nostril) of SARS-CoV-2 (hCoV-19/France/GE1973/2020, clade G, D614G (S)). This was considered day 0 post-infection (0 dpi). Non-infected animals received the same volume of PBS. At 1, 2, 3, and 4 dpi animals were treated intranasally as described above under inhalation anaesthetics (4% isoflurane). Clinical signs, body weight and oropharyngeal swabs were recorded and collected every day after challenge, until the end of the experiment (5 dpi). At 5 dpi all animals were euthanised via intraperitoneal administration of sodium pentobarbital (not exceeding a dose of 60 mg/kg). Prior to this, the animals were anesthetized using inhalation anesthesia (4% isoflurane). Lungs, spleens, hearts, kidneys and livers were then collected for pathological analysis. Further, lung samples were extracted to quantify viral loads and examine the expression levels of inflammatory marker genes. To quantify virus titers, lung tissues were homogenized using bead disruption and centrifuged to remove cellular debris. Supernatants were serially diluted (fivefold) and endpoint titrations were performed on confluent VeroE6 cells. TCID_50_ values were calculated by the Reed-Muench method. Total RNA from lungs was isolated with the RNeasy Mini kit (Qiagen). Complementary DNA (cDNA) was synthesized from 1 µg of total RNA by reverse transcription (RT) using LunaScript RT SuperMix (New England Biolabs). Primer sequences used for quantitative real-time polymerase chain reaction (qPCR) are listed in Table 1.

**Table 1:** RT-qPCR primer sequences.

Primer	Sequence	References
hmstr_ActB_fw	GGC CAG GTC ATC ACC ATT	^54^
hmstr_ActB_rev	GAG TTG AAT GTA GTT TCG TGG ATG	
hmstr_MX2_fw	CCA GTA ATG TGG ACA TTG CC	^54^
hmstr_MX2_rev	CAT CAA CGA CCT TGT CTT CAG TA	
hmstr_CCL5_fw	TCA GCT TGG TTT GGG AGC AA	^33^
hmstr_CCL5_rev	TGA AGT GCT GGT TTC TTG GGT	
hmstr_CXCL10_fw	GCC ATT CAT CCA CAG TTG ACA	^55^
hmstr_CXCL10_rev	CAT GGT GCT GAC AGT GGA GTC T	
hmstr_RIG1_fw	GTG CAA CCT GGT CAT TCT TTA TG	^55^
hmstr_RIG1_rev	GTC AGG AGG AAG CAC TTA CTA TC	
COV2_N2_fw	TTA CAA ACA TTG GCC GCA AA	^56^
COV2_N2_rev	GCG CGA CAT TCC GAA GAA	

### Immunohistochemical analysis

Lung, liver, heart, spleen, and kidney samples from hamsters were collected on day 5 after viral challenge, fixed by immersion in 10% buffered formalin and embedded into paraffin blocks. Transwell membranes from ALI cultures were fixed in 4% neutral-buffered formalin and embedded into paraffin blocks. Formalin-fixed paraffin-embedded tissue specimens and ALI cultures were sectioned at 3 µm. SARS-CoV-2 nucleocapsid protein was detected by immunohistochemical staining using the rabbit monoclonal antibody 40143-R019 (Sino Biological, Eschborn, Germany) at 1:500 dilution. For visualization of the immunolabelled cells, the EnVision® + detection system (Agilent, Santa Clara, CA, USA) with 3,3’-diaminobenzidine as peroxidase substrate was used.

### Evaluation of histopathological changes

Hematoxylin/eosin stained sections were examined by light microscopy and lung damage was semi-quantitatively scored based on the level of inflammation (0—no; 1—mild, 2 -moderate; or 3—severe) as described previously^52^.

### Statistical analysis

Statistical data analysis was performed in GraphPad Prism software version 9. Statistical significances were analyzed by one-way analysis of variances (ANOVA) followed by Bonferroni post hoc test to correct for multiple comparisons or by unpaired t-test. *P*-values < 0.05 were considered statistically significant.

## Electronic supplementary material

Below is the link to the electronic supplementary material.

Supplementary Material 1

Supplementary Material 2

## Data Availability

SARS-CoV-2 genome sequencing data from stock preparations used for in vitro experiments are available in the NCBI Sequence Read Archive (SRA) with the BioProject accession number: PRJNA1216935 (available from 2026-02-27). Raw data files are available from the corresponding authors upon reasonable request.
