# Supplementary material for: An ACE2-Fc decoy produced in glycoengineered plants neutralizes ancestral and newly emerging SARS-CoV-2 variants and demonstrates therapeutic efficacy in hamsters
Source: Sci Rep. 2025 Apr 2;15:11307. doi: 10.1038/s41598-025-95494-w (PMC11965572; doi:10.1038/s41598-025-95494-w)
Supplement: Supplementary file 2 — Supplementary Material 2 [file 41598_2025_95494_MOESM2_ESM.pdf]

An ACE2-Fc decoy produced in glycoengineered plants neutralizes ancestral and newly emerging SARS-CoV-2 variants and demonstrates therapeutic efficacy in hamsters

Esther Föderl-Höbenreich[1*], Shiva Izadi[2*], Lara Hofacker[2], Nikolaus F. Kienzl[2], Alexandra Castilho[2], Richard Strasser[2], Ferran Tarrés-Freixas[3,4], Guillermo Cantero[3,4], Núria Roca[3,4], Mònica Pérez[3,4], Cristina Lorca-Oró[3,4], Carla Usai[3,4], Joaquim Segalés[4,5], Júlia Vergara-Alert[3,4], Lukas Mach[2**], Kurt Zatloukal[1**]

[1] Diagnostic and Research Institute of Pathology, Medical University of Graz, Graz, Austria

[2] Institute of Plant Biotechnology and Cell Biology, Department of Biotechnology and Food Sciences, BOKU University, Vienna, Austria

[3] IRTA, Animal Health, Centre de Recerca en Sanitat Animal (CReSA), Campus de la Universitat Autònoma de Barcelona (UAB), 08193 Bellaterra, Catalonia, Spain

[4] Unitat mixta d'investigació IRTA-UAB en Sanitat Animal, Centre de Recerca en Sanitat Animal (CReSA), Campus de la Universitat Autònoma de Barcelona (UAB), 08193 Bellaterra, Catalonia, Spain

[5] Departament de Sanitat i Anatomia Animals, Facultat de Veterinària, Universitat Autònoma de Barcelona, 08193 Bellaterra, Catalonia, Spain

* Equally contributing authors

** Corresponding authors

E-Mail: kurt.zatloukal@medunigraz.at; lukas.mach@boku.ac.at

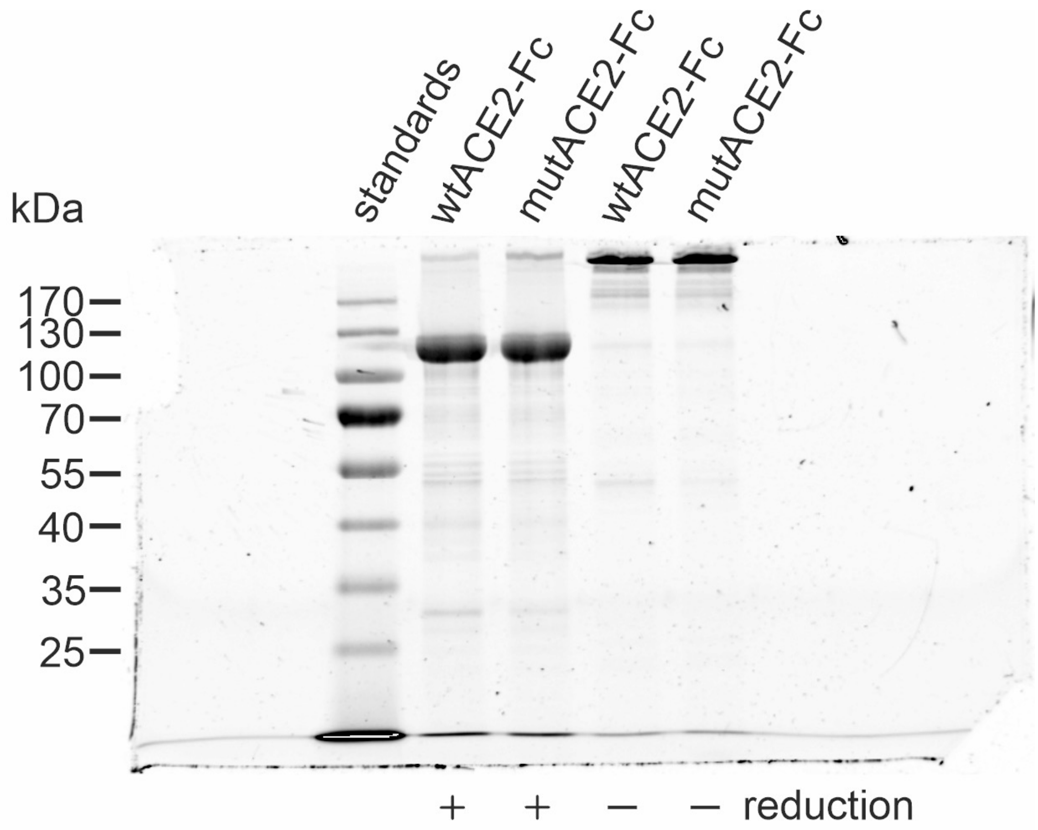

Supplementary Figure 1 SDS-PAGE analysis of purified wild-type (wt) and mutant (mut) ACE2-Fc under reducing and non-reducing conditions followed by Coomassie Brilliant Blue staining (uncropped gel of Figure 1a).

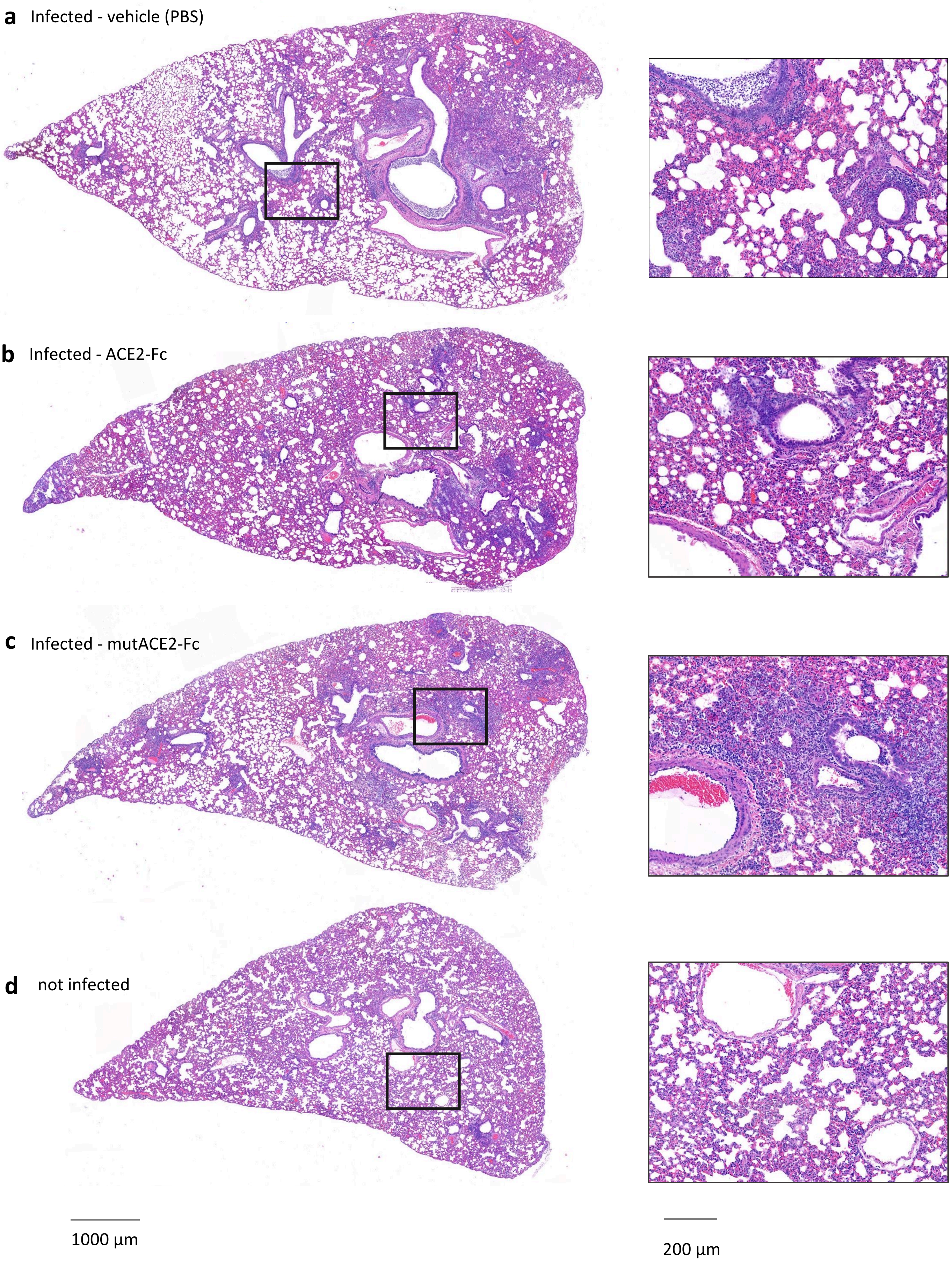

Supplementary Figure 2: Hematoxylin/eosin stained sections of lung tissues 5 days after infection. Lungs from animals treated daily with vehicle (a), wild-type ACE2-Fc (b) or mutACE2-Fc (c). Lung tissue of an uninfected animal (d) was used as a negative control. Representative sections are shown in two magnifications.
